# Plant-mediated gene silencing restricts growth of the potato late blight pathogen *Phytophthora infestans*

**DOI:** 10.1093/jxb/erv094

**Published:** 2015-03-18

**Authors:** Sultana N. Jahan, Anna K. M. Åsman, Pádraic Corcoran, Johan Fogelqvist, Ramesh R. Vetukuri, Christina Dixelius

**Affiliations:** ^1^ Swedish University of Agricultural Sciences, Department of Plant Biology, Uppsala BioCenter, Linnean Center for Plant Biology, P.O. Box 7080, SE-75007 Uppsala, Sweden; ^2^ Department of Evolutionary Biology, Uppsala University, Uppsala, Sweden

**Keywords:** Late blight, oomycete, *Phytophthora infestans*, potato, RNA interference, small RNA.

## Abstract

*Phytophthora infestans* is an oomycete that causes severe damage to potato, and is well known for its ability to evolve rapidly in order to overcome resistant potato varieties. An RNA silencing strategy was evaluated here to clarify if small interfering RNA homologous to selected genes in *P. infestans* could be targeted from the plant host to reduce the magnitude of the infection. As a proof-of-concept, a hairpin RNA (hp-RNA) construct using the *GFP* marker gene was designed and introduced in potato. At 72 hpi, a 55-fold reduction of the signal intensity of a corresponding GFP expressing *P. infestans* strain on leaf samples of transgenic plants, compared with wild-type potato, was detected. This suggests that an RNA interference construct in the potato host could be processed and target a transcript of the pathogen. Three genes important in the infection process of *P. infestans*, *PiGPB1*, *PiCESA2*, and *PiPEC*, together with *PiGAPDH* taking part in basic cell maintenance were subsequently tested using an analogous transgenic strategy. Out of these gene candidates, the *hp-PiGPB1* targeting the G protein β-subunit (*PiGPB1*) important for pathogenicity resulted in most restricted disease progress. Further, Illumina sequencing of inoculated transgenic potato leaves revealed sRNAs of 24/25 nt size homologous to the *PiGPB1* gene in the transgenic plants indicating post-transcriptional silencing of the target gene. The work demonstrates that a host-induced gene-silencing approach is functional against *P. infestans* but is highly dependent on target gene for a successful outcome. This finding broadens the arsenal of control strategies to this important plant disease.

## Highlight

A host-induced gene-silencing strategy for controlling potato late blight is presented, a plant disease that conventionally requires regular application of fungicides at high rates.

## Introduction

*Phytophthora infestans* is the oomycete pathogen responsible for the late blight disease on its potato host (*Solanum tuberosum*) inciting the worldwide most severe potato losses (Haverkort *et al.*, 2008; Forbes, 2012). Enormous breeding efforts to produce new varieties with improved resistance have been ongoing for more than 100 years. Exploitation of resistance genes from wild *Solanum* species started with *S. demissum* (Reddick, 1928, 1934) and has continued ever since (Vleeshouwers *et al.*, 2011). Besides pyramiding dominant resistance genes, the emphasis has been on introducing quantitative resistance traits to reduce the short ‘shelf life’ of qualitative resistance genes. However, finding durable resistance gene combinations without negative trait drag from donor species such as, for example, late maturity, remains a challenge. The success of *P. infestans* as a pathogen originates from its effective reproduction in both asexual and sexual forms. Under ideal conditions, the life cycle can be completed on foliage in about five days, where one lesion can generate up to hundreds of thousands of new sporangia (Fry, 2008). Furthermore, the genome of *P. infestans* is one of the largest among oomycetes (240Mb), containing vast numbers of transposable elements (TE) (Haas *et al.*, 2009). There are also hundreds of predicted genes coding for disease-promoting effector proteins, predominantly located in the TE-rich genomic regions which together drive the diversification process, all leading to an exceptional high potential to adapt to new control strategies of the potato crop.

Effector proteins in *P. infestans* belong mainly to two groups that target distinct sites in the host plant. The apoplastic effectors are secreted into the plant extracellular space, whereas cytoplasmic effectors are translocated inside the plant cell where they target different subcellular compartments (Armstrong *et al.*, 2005; Kamoun, 2006; Whisson *et al.*, 2007). Both classes of effectors are modular proteins with cleavable amino-terminal secretion signals. Cytoplasmic effectors carry an additional domain after the signal peptide that mediates translocation inside host cells and is defined by conserved motifs, such as the RXLR amino acid sequence. The discovery of many potential effectors in the genomic sequence of *P. infestans* has enabled high-throughput analysis for additional resistance gene candidates in a variety of species in the Solanaceae (Vleeshouwers *et al.*, 2008; Oh *et al.*, 2009).

Gene silencing or RNA interference (RNAi) is a master regulatory mechanism with diverse roles such as the control of gene expression at transcriptional and post-transcriptional levels and chromatin organization of eukaryotic organisms. Central players in RNA silencing are small RNAs (sRNAs), often ranging in size from 19–40 nucleotides, and divided into different classes with diverse roles (Ghildiyal and Zamore, 2009; Malone and Hannon, 2009; Ruiz-Ferrer and Voinnet, 2009). Generally, sRNAs are generated from long double stranded RNA precursors which are digested by the type III RNase called Dicer or Dicer-like (Dcl) into short duplexes known as siRNA (short interfering RNA). The duplex is unwound and the antisense strand incorporated to an Argonaute (Ago) protein, which then binds to homologous mRNA and degrades it or inhibits its translation. This intramolecular hybridization of self-complementary RNA based on hairpin or stem-loop RNA is a key step in RNA silencing. Another important protein associated with RNAi is RNA-dependent RNA polymerase which is responsible for the conversion of RNA into double-stranded RNA and for the amplification of the silencing signal through the generation of secondary siRNA.

*P. infestans* possesses the components of canonical gene silencing pathways similar to those of other eukaryotes but the involved proteins display unusual protein domain organization (Vetukuri *et al.*, 2011a). First, only one Dicer-like protein, having the expected dual RNase III domains but lacking other typical domains for Dicers, was identified. A second Dicer-like protein (PiDcl2) was later found in the genome trace archive (Fahlgren *et al.*, 2013). Small RNAs of approximately 21 nt have been associated with partial silencing in *P. infestans* (Ah-Fong *et al*., 2008) and 40 nt sRNAs were associated with TE silencing (Vetukuri *et al.*, 2011b). Based on deep sequencing of sRNAs from *P. infestans*, distinct classes of sRNAs at 21, 25/26, and 32 nt were found where the biogenesis of 21 nt sRNAs was shown to be PiDcl1-dependent while longer sRNAs were PiAgo-dependent (Vetukuri *et al.*, 2012). Furthermore, *P. infestans* lacks DNA methylation associated proteins known to be ubiquitous in plants.

Plants utilize post-transcriptional gene silencing to protect themselves against invasive nucleic acids such as transposons, viruses, and transgenes (Cogoni and Macino, 2000). Knowledge on the basic mechanisms of gene silencing provides new opportunities to explore plant–pathogen interactions and potential strategies for novel disease control. In this area there are promising reports where RNAi-based constructs in plants were designed to target fungal plant pathogens (Nowara *et al.*, 2010; Koch *et al.*, 2013; Ghag *et al.*, 2014). In order to expand the toolbox for potato breeders not just to rely on dominant resistance genes from various *Solanum* species, it would be interesting to know if a similar gene-silencing approach driven by the plant host could be a functional strategy to target this oomycete plant pathogen.

The main objective of this study was to investigate the possibility of exploiting RNA silencing to target selected genes in *P. infestans* via the host plant and, thereby, reduce its capacity to initiate and develop disease on potato. The work showed that sRNAs targeting a pathogen gene, generated in the host plant during infection, incites host-induced gene silencing of the corresponding pathogen transcript. However, the choice of target genes and precursor hp-RNA is crucial for a successful outcome of this strategy.

## Materials and methods

### *P. infestans* strains and plant inoculation

The *eGFP* strain under the promoter of *Ham34* (Avrova *et al.*, 2008) and the wild-type 88069 strain were cultured as described earlier by Vetukuri *et al.* (2011a). Each *P. infestans* strain at a concentration of 5×10^4^ spores ml^–1^ water was used for leaf inoculations (Grenville-Briggs *et al.*, 2005) of potato plants at 4 weeks post transfer to soil from *in vitro* conditions.

### Potato transformation and cultivation

For transformation, *in vitro* potato plants of cv. Desiree were grown at 22 °C with a 16h photoperiod on Murashige–Skoog (MS) medium (Duchefa Biochemie B.V., Amsterdam, Netherlands), supplemented with 2% (w/v) sucrose and 0.3% (w/v) gelrite (Duchefa). The internodes of the plants were used as explants for transformation (Millam, 2007) using the *Agrobacterium tumefaciens* strain C58. Ten potential transgenic shoots per construct were grown on MS media with 50 μg ml^–1^ kanamycin. Plantlets were transferred to soil and grown under the same light and temperature conditions in a culture chamber as for the plants grown *in vitro*. Validation of transgenic plants was done by PCR (the primers are listed in Supplementary Table S1 at *JXB* online) and Sanger sequencing.

### hp-RNA constructs for potato transformation

All the gene-silencing constructs were made with the ubiquitin1 (*UBQ1*) promoter for constitutive expression and the heat shock protein 18.2 (*HSP*) terminator from *Arabidopsis thaliana.* To enable a hairpin (hp) formation of selected sequences, a 71bp intron from the Ste20-like *PiC20* gene of *P. infestans* (Tani *et al.*, 2004) was used. cDNA of 88069 and the *Ham34:eGFP* strains of *P. infestans* were used as templates in the gene PCR amplification step. Besides the green fluorescence protein (*GFP*) marker gene, sequences of the following genes were used: G-protein β-subunit (*PiGPB1*) (Latijnhouwers and Govers, 2003), cellulose synthase A2 (*PiCESA2*) (Grenville-Briggs *et al.*, 2008), pectinesterase (*PiPEC*) (Ospina-Giraldo *et al.*, 2010a), and the constitutive glyceraldehyde 3-phosphate dehydrogenase (*PiGAPDH*) gene. The choice of target genes relied on published data on *PiCESA1* and *PiGAPDH* (Zeng *et al.*, 2006; Grenville-Briggs *et al.*, 2008) together with our analysis of *PiGPB1* and *PiPEC* in infected potato plants (see Supplementary Fig. S1 at *JXB* online). The specificity of each target gene was determined by BLAST and, using sequences from highly conserved nucleotides, from CLUSTAL-W alignments within each gene family. The BP and LR reactions were performed by using MultiSite Gateway® Pro Technology (Invitrogen, Carlsbad, CA, USA), and recombined into the binary vector pGWB1 (Nakagawa *et al.*, 2007). All primers used for plasmid construction are listed in Supplementary Table S2 at *JXB* online, and detailed cloning steps are outlined in Supplementary Fig. S2 at *JXB* online. All constructs were confirmed by sequencing (Macrogen Inc. Seol, Korea).

### Quantitative real-time PCR (qRT-PCR)

Leaf samples from wild-type, cv. Desiree, and transgenic plants were collected at 24, 48, and 72h post-inoculation (hpi), snap-frozen in liquid nitrogen, and stored at –70 °C. The leaf materials were used for RNA extraction, cDNA synthesis, and SYBR green qRT-PCR assays (Vetukuri *et al.*, 2011a). To check the transcript level for each gene of interest, specific primers were designed to anneal outside the hairpin sequence. Primers are listed in Supplementary Table S3 at *JXB* online. Calculations and statistical analyses were carried out according to Avrova *et al.* (2003). Primers for hairpin expression analysis are listed in Supplementary Table S4 at *JXB* online. DNA quantification of *P. infestans* and calculation was as described earlier by Llorente *et al.* (2010). At least three biological replicates were used for each individual qRT-PCR analysis.

### Phenotypic analysis of transgenic potato plants

Colonization and disease progression were monitored up to 30 d after inoculations. The effect of *PiGPB1* silencing on spore formation was assayed by analysing the shape and number of sporangia using an epifluorescence microscope (Leica DMI 4000). Sporangia were collected at 24, 48, 72, and 96 hpi from detached leaves and counted 1h after leaf detachment as described by Lehtinen *et al.* (2009).

### Confocal microscopy

The *GFP* expression levels on leaves of the transgenic and wild-type plants inoculated by the *GFP*-expressing *Ham34:eGFP P. infestans* strain were analysed by confocal microscopy (Zeiss LSM510). GFP was excited at 488nm,and detected at 505–530nm. Images were analysed using the LSM 510 software.

### Northern blot hybridization

To detect sRNA molecules homologous to the *PiGPB1* gene, Northern blot hybridization was performed (Vetukuri *et al.*, 2011b). Primers used for the generation of *PiGPB1-*specific riboprobes were: Spe-Fw ATATACTAGTAGT TCTCAGCCAATCTTCG and Not-Rev ATATGCGGCCGCTTCAACTTGGT CTAGTTTCCAT.

### Western blotting

Total GFP proteins from the *GFP*-tagged *P. infestans* strain colonized on hp-GFP transgenic plants, were resolved on SDS-containing 9% polyacrylamide gel. The proteins were transferred to nitrocellulose membrane and incubated with an anti-GFP monoclonal antibody, following the procedure described previously by Moschou *et al.* (2008). The membranes were subsequently incubated with a chemi-luminescent substrate (ECL Western blotting system kit, GE Healthcare, Uppsala) and exposed using a Fuji Phosphorimager. Ponceau staining of the membrane (Bannur *et al.*, 1999) was used as a loading control.

### Small RNA sequencing

Total RNA was extracted from hp-PiGPB1 transgenic and wild-type potato leaves at 24, 48, and 72 hpi infected by the 88069 strain, the 88069 mycelia control, and non-infected transgenic plants using the mirVana^TM^ miRNA isolation kit (Ambion, Austin, TX, USA). Eight sRNA libraries were generated using the Illumina small RNA sample preparation kit and sequenced using Illumina HiSeq 2500 at SciLifeLab, Stockholm, Sweden.

### Bioinformatic analysis

All sRNA reads were trimmed at any base with a phred-scaled quality score below 10 and adapter sequences were removed using Cutadapt v1.3 (Martin, 2011). Reads shorter than 18 bases in length were excluded from all further analysis. The resulting set of filtered reads for each sample was aligned to the *P. infestans* reference genome (http://www.broadinstitute.org/annotation/genome/phytophthora_infestans/MultiDownloads.html) using Bowtie2 v2.1.0 (Langmead and Salzberg, 2012) with one mismatch allowed per seed (-N 1), a seed length of 18 (-L 18), and the remainder of alignment options set to their default values. The SAM format alignments produced by Bowtie2 were converted to BAM files, sorted, and indexed using SAMtools v0.1.19 (Li *et al.*, 2009). The number, locations, lengths, and sense/antisense orientations of reads mapping to the *PiGPB1* gene in *P. infestans* were obtained using a combination of the SAMtools view command and custom python scripts.

## Results

### Generation and validation of transgenic potato harbouring hp-RNA constructs

A series of gene-cloning steps were performed to accomplish binary vectors containing the target gene sequence in a sense and antisense orientation together with an intron (I) to enable a short hp-RNA formation (see Supplementary Fig. S2 at *JXB* online). This construction work resulted in five binary vectors: *UBQ1:GFP-I-GFP*; *UBQ1:PiGPB1-I-PiGPB1*; *UBQ1:PiCesA2-I-PiCesA2*; *UBQ1:PiPEC-I-PiPEC*; and *UBQ1:PiGAPDH-I-PiGAPDH*, that were used for *A. tumefaciens*-mediated transformation. Ten transgenic potato plants from each construct were made, and three individual lines L1, L2, and L3 were used in subsequent analysis. The transgenic status of the plant materials was validated by PCR amplification of the sequences matching individual constructs in DNA samples from the different transgenic plants (see Supplementary Fig. S3A at *JXB* online) and by performing Sanger sequencing to check that each construct was intact in the plants, followed by analysing the transcription levels of each gene by qRT-PCR (see Supplementary Fig. S3B at *JXB* online). Further, in order to verify that the silencing of the target gene was not an effect of the transformation procedure it self, the *PiGPB1* transcript level was analysed in hp-GFP plants and compared with hp-PiGPB1 and wild-type potato plants infected with *P. infestans*. The *PiGPB1* transcript level in the hp-GFP plants compared with wild-type plants remained intact at 24, 48, and 72 hpi while the transcript level was gradually decreasing with time in hp-PiGPB1 plants as expected (see Supplementary Fig. S3C at *JXB* online).

### A *GFP*-expressing *P. infestans* strain can be targeted from the host plant

**Fig. 1.**
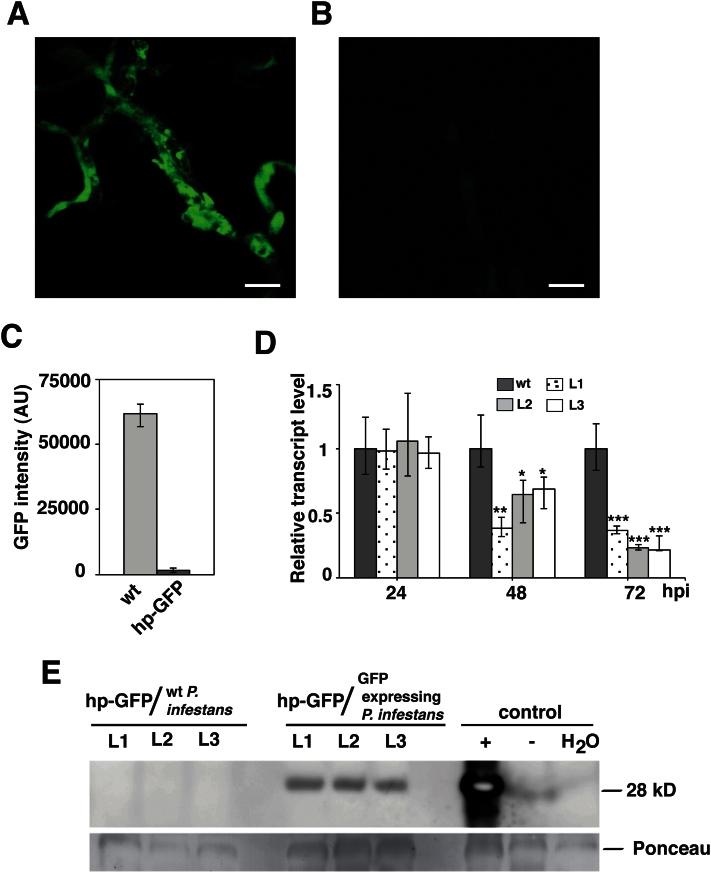
Silencing of *GFP* in *P. infestans* by hp-RNA. Confocal laser scanning microscopy of *P. infestans* transformants (*Ham34:eGFP*) expressing green fluorescent protein. *GFP* expression in mycelia grown on (A) wild-type and (B) hp-GFPL1 (*UBQ:GFP-I-GFP*) transgenic potato. Bars=25 μm. (C) Fold change of total intensity of GFP in mycelia grown on hp-GFP plants compared with wild-type plants. AU=arbitrary unit. (D) Transcript abundance of *GFP* in *P. infestans* transformants grown on wild-type and transgenic plants at 24, 48, and 72 hpi, quantified by qRT-PCR. Data are normalized to *P. infestans actinA* mRNA levels and represent means ±SE (*n*=3 pooled leaves of 3 plants). Asterisks indicate significant difference to the wild type (Student’s *t* test; *P <0.05; **P <0.01; ***P <0.001). (E) Western blot analysis of GFP protein. hp-GFP plant lines inoculated with: wild type (wt) *P. infestans* (88069), the GFP-tagged *P. infestans*, wild-type plants inoculated with: the GFP-tagged *P. infestans* (+), 88069 (–), and water. Ponceau staining was used as loading control.

Three transgenic potato lines (hp-GFPL1, hp-GFPL2, and hp-GFPL3) harbouring the hp-RNA construct matching a *GFP*-tagged *P. infestans* strain were analysed first. The intensity of the GFP signal in the *P. infestans-*colonizing transgenic potato leaves was monitored and quantified using confocal microscopy. There was a marked reduction in the GFP signal at 72 hpi in all three transgenic lines compared with wild-type plants (Fig. 1A, B; see Supplementary Figs S4 and S5 at *JXB* online). At 72 hpi, as much as a 55-fold reduction of the signal intensity of the *GFP-*expressing *P. infestans* strain was recorded on leaf samples of transgenic plants compared with wild-type potato (Fig. 1C). In order to monitor the *GFP*-silencing in *P. infestans* further, transcript levels were checked. A significant reduction in relative transcript levels of the *GFP* gene in *P. infestans* growing on transgenic plants at 48 and 72 hpi was detected compared with wild-type potato (Fig. 1D). To check if the protein level in the hp-GFP potato lines was altered, a Western blot was run (Fig. 1E). A reduction of the GFP protein was seen in the potato transgenic lines compared with wild-type plants upon infection with the *GFP*-tagged *P. infestans* isolate. Together, these data suggest that hp-RNA in the potato host could be processed and target a transcript of the invading *P. infestans* pathogen.

### Monitoring RNA-mediated inhibition of disease progression

**Fig. 2.**
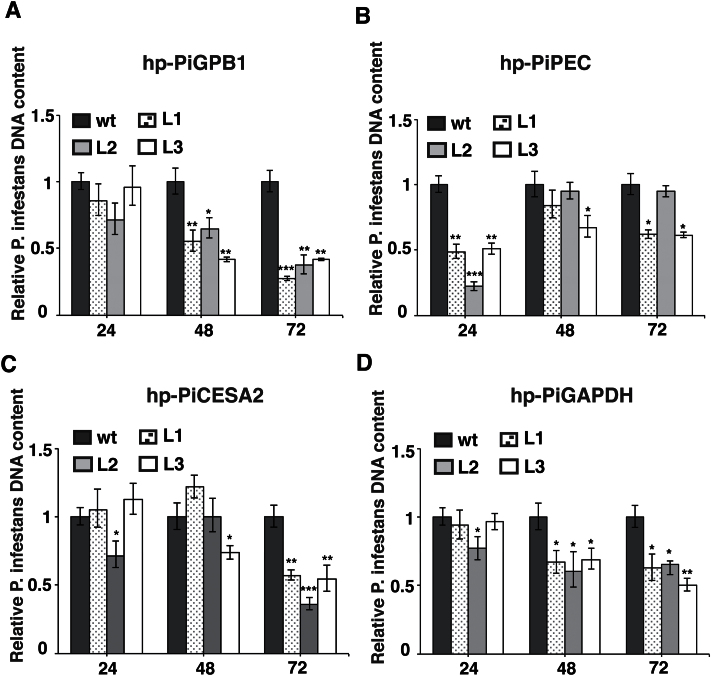
*P. infestans* DNA in leaves of the wild type and three individual transgenic lines L1, L2, and L3 of (A) hp-PiGPB1, (B) hp-PiPEC, (C) hp-PiCESA2, and (D) hp-PiGAPDH potato plants quantified by qRT-PCR at 24, 48, and 72 hpi. Data are normalized to potato *EF1* DNA levels and represent means ±SE (*n*=3 pooled leaves of 3 plants). Asterisks indicate significant difference to the wild type. (Student’s *t* test; *P <0.05; **P <0.01; ***P <0.001).

Encouraged by the data on *GFP* silencing in *P. infestans*, the next step was to monitor the effects of host-mediated silencing of the *PiGPB1*, *PiCESA2*, *PiPEC*, and *PiGAPDH* genes. A reduction of *P. infestans* DNA content over time on these transgenic plant leaves compared with the wild type was generally found but that trend was most obvious in the hp-PiGPB1 lines (Fig. 2A). In the case of the hp-PiPEC plants, a rather rapid decrease of *P. infestans* DNA was already detected at 24 hpi (Fig. 2B). By contrast, the DNA levels were lowest at 72 hpi compared with earlier time-points in the hp-PiCESA2 plants but not reaching the level seen in the hp-PiGPB1 lines (Fig. 2C). In the leaf samples from the hp-PiGAPDH transgenic lines, the growth of *P. infestans* seemed to be less at 48 and 72 hpi compared with 24 hpi (Fig. 2D). For all these transgenic plants, colonization by *P. infestans* occurred as necrotic lesions on the leaves to a limited extent compared with the wild-type plants (see Supplementary Fig. S6 at *JXB* online).

### Reduced transcript level in hp-PiGPB1 and hp-PiGAPDH plants

**Fig. 3.**
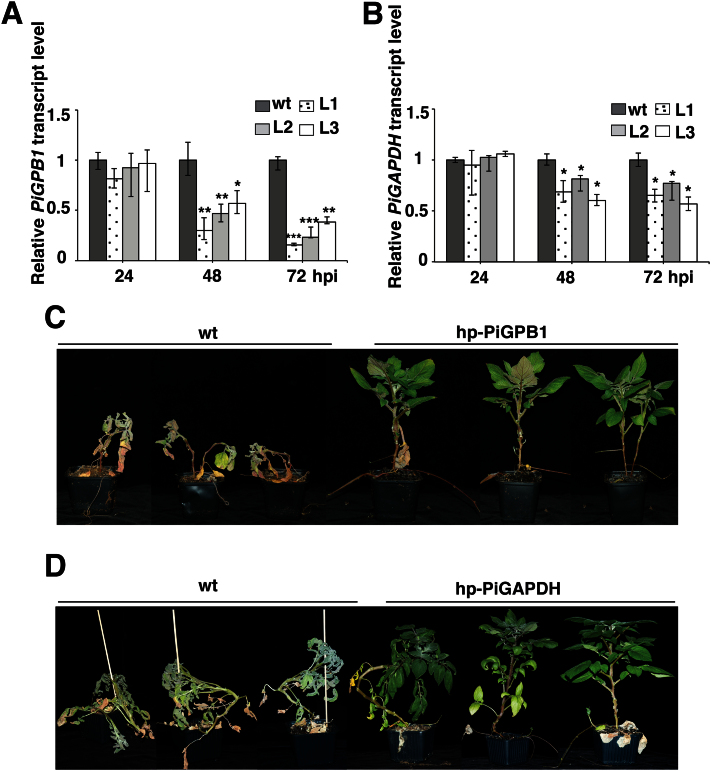
Analysis of transgenic potato harbouring a *hp-PiGPB1* or *hp-PiGAPDH* construct. (A) Transcript abundance of the *PiGPB1* in wild-type and three lines of hp-PiGPB1 plants. (B) Transcript abundance of the *PiGAPDH* gene in wild-type and hp-PiGAPDH plants inoculated with *P. infestans* at 24, 48, and 72 hpi quantified by qRT-PCR. Data are normalized to *P. infestans actinA* mRNA levels and represent means ±SE (*n*=3 pooled leaves of 3 plants). Asterisks indicate significant difference to the wild type (Student’s *t* test; *P <0.05: **P <0.01; ***P <0.001). Overall performance of (C) wild-type and hp*-*PiGPB1 transgenic plants, and (D) wild-type and hp*-*PiGAPDH transgenic plants at 30 dpi.

Further studies were done on the hp-PiGPB1 plants where a gene important for sporangia formation was targeted with promising effects on the pathogen. The basic cell maintenance gene in the hp-PiGAPDH plants was included for comparison. In order to monitor the silencing of *PiGPB1* and *PiGAPDH* in *P. infestans*, transcript levels of these genes were checked in the transgenic material. The relative transcript levels of *PiGPB1* at 48 and 72 hpi were significantly down-regulated in all three transgenic lines compared with inoculated wild-type plants (Fig. 3A). In the case of the hp-PiGAPDH plants, a reduced transcript level of *PiGAPDH* was particularly seen at 48 hpi and 72 hpi (Fig. 3B). These results were in line with a reduced *P. infestans* DNA content detected in infected leaves. To record overall plant performance, the plants were allowed to grow for a month. After 30 d, the inoculated wild-type plants were almost dead, whereas transgenic hp-PiGPB1 plants showed greatly reduced disease spread disease symptoms (Fig. 3C). A similar performance was seen for the hp-PiGAPDH transgenic plants (Fig. 3D), although the effect was not as prominent compared with the hp-PiGPB1 plants.

### Host induced silencing of *PiGPB1* gene affects spore formation

**Fig. 4.**
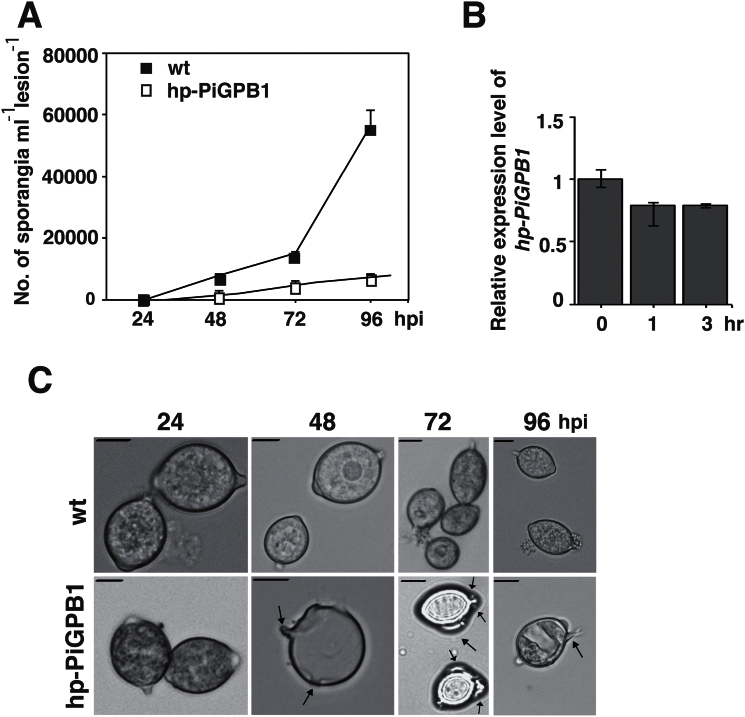
Effects on spore formation in transgenic hp-PiGPB1 plants. (A) Numbers of sporangia produced by the 88069 strain of *P. infestans* on wild-type and transgenic plants at 24, 48, 72, and 96 hpi. Data represent means ±SE (*n*=3 pooled samples of 3 plants). (B) Relative expression of *hp-PiGPB1* construct in hp-PiGPB1 plants inoculated with *P. infestans* at 0, 1, and 3h after leaf detachment. Data are normalized to *P. infestans actinA* mRNA levels and represent means ±SE (*n*=3 pooled leaves of 3 plants) (C) Phenotypes of sporangia collected at 24, 48, 72, and 96 hpi from wild-type and hp-PiGPB1 transgenic plants. Bars =10 μm.

*PiGPB1* is a gene known to be involved in phosphatase-mediated signalling in mycelia leading to the formation of sporangia (Kasahara *et al.*, 1997; Latijnhouwers and Govers, 2003). Silencing of this gene causes *P. infestans* to produce abundant aerial mycelia and very few defective sporangia. In the present study, no sporangia were found on leaves of the hp-PiGPB1 plants. In order to assay any phenotypic changes whatsoever, a detached leaf assay was used where inoculated leaves were taken at 24, 48, 72, and 96 hpi from the hp-PiGPB1 plants. Leaving the leaves detached for 1h in a plastic box under humid conditions promoted sporangia formation. A condition that by time favoured the pathogen over the plant host with declining *hp-PiGPB1* expression (Fig. 4B). Yet, a 6-fold reduction in the number of sporangia on colonized transgenic leaves was observed compared with the wild-type at 96 hpi (Fig. 4A). Sporangia produced by *P. infestans* at 24 hpi, grown on both control and hp-PiGPB1 leaves, were normal with the expected ‘lemon shapes’ and single hyphae. By contrast, sporangia formed on hp-PiGPB1 plants at 48, 72, and 96 hpi were abnormal with an outgrowth of multiple hyphae and the loss of the characteristic phenotype at 48 hpi (Fig. 4C). This reduction in number of sporangia and sporangia with multiple hyphal outgrowths are in agreement with the phenotype of the *PiGPB1*-silenced mutants of *P. infestans* observed earlier by Latijnhouwers and Govers (2003).

### Detection of sRNAs homologous to *PiGPB1* gene in *P. infestans*

**Fig. 5.**
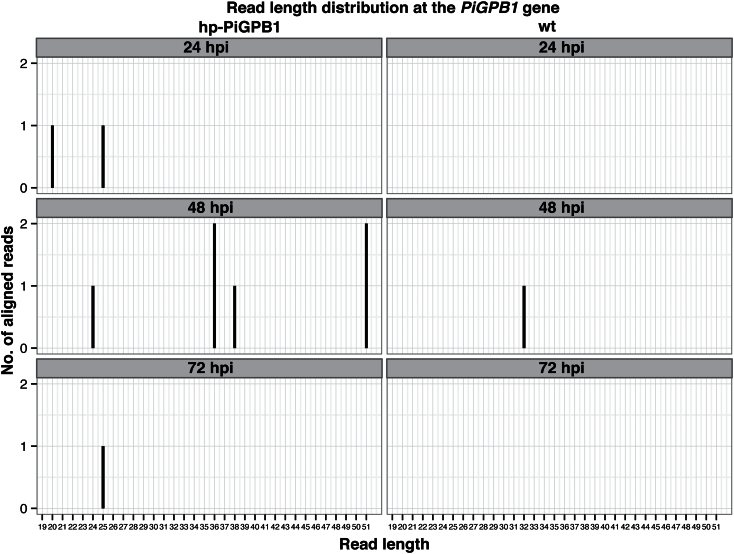
Small RNAs complementary to the *PiGPB1* gene. Distribution of small RNA reads homologous to the *PiGPBI* gene in transgenic hp-PiGPB1 and wild-type potato plants at 24, 48, and 72 hpi based on Illumina sequencing.

In order to confirm the presence of sRNAs homologous to hp-RNA constructs in transgenic plants, Northern blot hybridizations were used. Probes specific to *PiGPB1* detected antisense sRNAs, ranging from 21, 25, 26, 27, and 28 nt in size (see Supplementary Fig. S7 at *JXB* online). Subsequently, Illumina sequencing of sRNAs generated from wild-type and hp-PiGPB1 transgenic plants resulted in unique but few 24/25 nt size class sRNAs only mapping to the *PiGPB1* gene in the transgenic plants and not seen in control plants (Fig. 5). These data suggest that siRNAs are generated from the hp-RNA constructs in transgenic plants during the infection process.

## Discussion

This work demonstrated the possibility that hp-RNA constructs in potato could affect the host colonization and invasion by *P. infestans* to various degrees. Three genes of importance in the first stages of infection, together with a gene involved in basic cell maintenance, were examined. The current understanding of key genes and signals in early plant infection phases is meagre. It could be anticipated that various receptor classes are main players in these interactions, not least G protein-coupled receptors common in eukaryotes. Heterotrimeric G proteins are known to be key regulators in numerous signalling pathways impacting arrays of downstream targets. However distinct differences are known between animals and plants (Urano and Jones, 2014). *P. infestans* has only a single α (*PiGPA1*) and one β (*PiGPB1*) subunit gene. *PiGPB1* is differentially expressed in various stages of the life cycle of *P*. *infestans* (Laxalt *et al.*, 2002; Latijnhouwers and Govers, 2003; Dong *et al.*, 2004). Primarily, *PiGPB1* is important for sporangia formation and is not strongly expressed in mycelia. When the infection progresses from a biotrophic to a necrotrophic mode at 36–48 hpi, the continuous formation of sporangia is required (Judelson and Blanco, 2005), a process which is impaired in the hp*-*PiGPB1 transgenic plants. Fewer sporangia, and at the same time abnormal phenotypes, impose difficulties to infect new host cells leading to reduced colonization and spread of the pathogen.

Oomycetes have cell walls composed of cellulose, not chitin as fungi do, but both pathogenic organism groups have evolved pectinases to break down plant cell walls, enzymes aiding in the penetration and subsequent establishment of the infection. *Phytophthora* species encode large sets of different cell wall-degrading enzymes (Ospina-Giraldo *et al.*, 2010b). Thus, influencing cell wall-associated genes, such as hp-PiCESA2 and hp-PiPEC, was also of great interest. In the hp-PiCESA2 plants, *P. infestans* is supposed to have impaired the formation of appressoria (Grenville-Briggs *et al.*, 2008), leading to attenuated colonization but such an effect was not consistent over time. On the hp-PiPEC plants, *P. infestans* encountered difficulty in establishing the infection process but the few spores that managed to penetrate the mesophyll cells continued to colonize the leaves. Some of this ‘incomplete’ inhibition could be attributed to redundancy since *CESA*, *PEC*, and *GAPDH* belong to gene families of varying size in *P. infestans*. The challenge here was to design hp-gene constructs that specifically targeted *P. infestans* and not the host plant.

In the *Arabidopsis*–*Phytophthora parasitica* interaction, host-driven gene-silencing targeting *GFP* and the *PnPMA* gene encoding for a plasma membrane H^+^ ATPase were not reported to be successful and was explained by the lack of proper machinery required for the uptake of silencing signals in the oomycete pathogen (Zhang *et al.*, 2011). This is contrary to what is already a well-established methodology in oomycete research; experiments where the treatment of protoplasts with hp-RNA results in transient RNA silencing, which shows beyond doubt that oomycetes do have the machinery needed for the uptake of silencing molecules (Whisson *et al.*, 2005; Vetukuri *et al.*, 2011a). Nevertheless, the mechanism of RNA uptake from the plant host during the infection process into *P. infestans* cells has not been well studied. It was shown recently that the fungus *Botrytis cinerea* delivers sRNAs to hijack the host RNAi machinery and selectively silences host immunity genes during the infection process of *Arabidopsis* (Weiberg *et al.*, 2013). Notably, out of the 73 *B. cinerea* sRNAs with the potential to silence plant genes, 52 were derived from transposable elements (TE) of the fungal genome. If sRNAs are included in the vast list of *P. infestans* effectors, this implies thousands of new candidates because of its TE-saturated genome constitution, and adds to the enormous capacity to overcome host resistance. The Illumina sequencing data revealed sRNAs of 24/25 nt size homologous to the *PiGPB1* gene in the transgenic plants, indicating post-transcriptional silencing of the target gene. The number of sRNA reads detected were considerably less than expected which could be attributed to unknown timing for the required cellular processes, which thereby leads to non-optimal timing of sample collection. However, based on hairpin RNA expression studies in plants, it can be presumed that the sRNAs are initially formed on the plant side (Mansoor *et al.*, 2006).

The process of effector protein translocation between potato and *P. infestans* has been explored for many years and, hitherto, led to inconclusive results (Wawra *et al.*, 2013). Similarly, the sRNA translocation process is poorly understood. No homologous proteins to SID1 and SID2, earlier identified in *C. elegans* to be responsible for the intercellular communication of the RNA-induced silencing signal, and also environmental RNA interference (Winston *et al*., 2002, 2007) are neither found in *P. infestans* nor in potato or *Arabidopsis* genomes. In the case of the barley–*Blumeria graminis* interaction, exosomes have been speculated to be involved in the translocation of the silencing signal (Nowara *et al.*, 2010) but this critical question remains to be answered.

### Future potential

The hp-RNA-mediated gene silencing of *P. infestans* clearly contributes to reduce disease progress in potato and should be regarded as an additional strategy to complement resistance gene deployment. Broader resistance would most likely be achieved by combining several RNAi transgenes, preferably targeting single copy genes of importance in the infection process. Understanding potential sRNA transport from *P. infestans* to the host plant opens up alternative revenues to control this important plant pathogen. During the processing of this paper one work on the interaction between B. lcatucae and lettuce was published (Govindarajulu *et al*., 2014).

## Supplementary data

Supplementary data can be found at *JXB* online.

Supplementary Fig. S1. Data on *in planta* expression of *PiGPB1* and *PiPEC* gene.

Supplementary Fig. S2. Schematic representation of plasmid constructs.

Supplementary Fig. S3. Data on hp-RNA constructs.

Supplementary Fig. S4. Silencing of *GFP* in *P. infestans* by hp-RNA.

Supplementary Fig. S5. Mycelia growth in hp-GFP transgenic potato lines.

Supplementary Fig. S6. Overall picture of necrotic lesions on leaves.

Supplementary Fig. S7. Northern blot analysis.

Supplementary Table S1. PCR primers for transgene detection.

Supplementary Table S2. Primers for plasmid constructs.

Supplementary Table S3. Primers for qRT-PCR.

Supplementary Table S4. Primers for hp-RNA expression analyses.

Supplementary Data
