# Supplementary material for: Plant-mediated gene silencing restricts growth of the potato late blight pathogen Phytophthora infestans
Source: J Exp Bot. 2015 Mar 18;66(9):2785–94. doi: 10.1093/jxb/erv094 (PMC4986879; doi:10.1093/jxb/erv094)
Supplement: Supplementary Data [file supp_66_9_2785__index.html]

Plant-mediated gene silencing restricts growth of the potato late blight pathogen Phytophthora infestans — Plant-mediated gene silencing restricts growth of the potato late blight pathogen Phytophthora infestans — Supplementary Data 

# Plant-mediated gene silencing restricts growth of the potato late blight pathogen *Phytophthora infestans*

## Supplementary Data

Data files

**Files in this Data Supplement:**

- Supplementary Data - Supplementary Data
