# Supplementary material for: Plant-mediated gene silencing restricts growth of the potato late blight pathogen Phytophthora infestans
Source: J Exp Bot. 2015 Mar 18;66(9):2785–94. doi: 10.1093/jxb/erv094 (PMC4986879; doi:10.1093/jxb/erv094)
Supplement: Supplementary Data [file supp_erv094_jexbot141648_file001.pdf]

Plant-mediated gene silencing restricts growth of the potato late blight pathogen *Phytophthora infestans*

Sultana Jahan, Anna Åsman, Pádraic Corcoran, Johan Fogelqvist, Ramesh Vetukuri and Christina Dixelius

Supplementary Data

Supplementary Table S1. PCR primers for transgene detection (Supplementary Figure S3A)

Gene	Primer name	Primer sequence (5´-3´)	Product size
PiGPB1 (PITG_06376.1)	gpbAS_F<br>gpbAS_R	TGGTCTTGCCCGTTCTGATA<br>CAAGATCTACGCCATGCAAT	491
PiPEC (PITG_02545.1)	pecAS_F<br>pecAS_R	CGGCACCATTAATGTATGACTT<br>TAGCACGCTCTTCTTGATGC	479
PiCESA2 (PITG_09964.1)	ces_AS_F<br>ces_AS_R	CGGCACCATTAATGTATGACTT<br>TAGCACGCTCTTCTTGATGC	451
PiGAPDH (PITG_17130)	pecS_F<br>pecS_R	AGCACCTCCTTCTCCATCAA<br>CTGCACAACTTTGTCTGCAAG	155
GFP	gfpAS_F<br>gfpAS_R	GTTCACCTTGATGCCGTTCT<br>CTGGACGGCGACGTAAAC	441
EF1a (AB061263.1)	EF1_F<br>EF1_R	TGAGGCAAACTGTTGCTGTC<br>TGGAAACACCAGCATCACAC	161

Supplementary Table S2. Primers for plasmid constructs

Gene	Primer name	Primer sequence (5´-3´)
UBQ-1 (*At3g62250*)	attB1UBQ_F	GGGGACAAGTTTGTACAAAAAAGCAGGCTCA TCTGAACAGCTTTGGATCTTTT
	attB5rUBQ_R	GGGGACAACTTTTGTATACAAAGTTGT TTTGTGTTTCGTCTTCTCTCAC
HSP 18.1 (*At5g59720*)	attB3HSP_F	GGGGACAACTTTGTATAATAAAGTTGTC ATATGAAGATGAAGATGAAA
	attB2HSP_R	GGGGACCACTTTGTACAAGAAAGCTGGGTA CTTATCTTTAATCATATTCC
GFP	gfp sense_F	ATATGGCCGGCCCTGGACGGCGACGTAAAC
	gfp sense _R	ATATCCTGCAGGGTTCACCTTGATGCCGTTCT
	attB5gfpS_F	GGGGACAACTTTGTATACAAAAGTTGCA CTGGACGGCGACGTAAAC
	attB4 gfpS_R	GGGGACAACTTTGTATAGAAAAGTTGGGTG CTAAAGAATCGCCACGTGAGT
	attB4rgfpAS_F	GGGGACAACTTTTCTATACAAAGTTGCA GTTCACCTTGATGCCGTTCT
	attB3rgfpAS_R	GGGGACAACTTTATTATACAAAGTTGT CTGGACGGCGACGTAAAC
PiGPB1 (*PITG_06376.1)*	gpbS_F	ATATGGCCGGCCCAAGATCTACGCCATGCAAT
	gpbS_R	ATATCCTGCAGGCTAAAGAATCGCCACGTGAGT
	attB5gpbS_F	GGGGACAACTTTGTATACAAAAGTTGCA CAAGATCTACGCCATGCAAT
	attB4gpbS_R	GGGGACAACTTTGTATAGAAAAGTTGGGTG CTAAAGAATCGCCACGTGAGT
	attB4rgpbAS_F	GGGGACAACTTTTCTATACAAAGTTGCA TGGTCTTGCCCGTTCTGATA
	attB3rgpbAS_R	GGGGACAACTTTATTATACAAAGTTGT CAAGATCTACGCCATGCAAT
PiCESA2 (*PITG_09964.1)*	ces_F	ATATGGCCGGCCTAGCACGCTCTTCTTGATGC
	ces_R	ATATCCTGCAGGTAAAGAATCGCCACGTGAGT
	attB5ces S_F	GGGGACAACTTTGTATACAAAAGTTGCA TAGCACGCTCTTCTTGATGC
	attB4ces S_R	GGGGACAACTTTGTATAGAAAAGTTGGGTG CTAAAGAATCGCCACGTGAGT
	attB4rcesAS_F	GGGGACAACTTTTCTATACAAAGTTGCA CGGCACCATTAATGTATGACTT

	attB3rcesAS_R	GGGGACAACTTTATTATACAAAGTTGT TAGCACGCTCTTCTTGATGC
PiPEC (PITG_02545.1)	pecS_F	ATATGGCCGGCC TAGCACGCTCTTCTTGATGC
	pecS_R	ATATCCTGCAGGTAAAGAATCGCCACGTGAGT
	attB5pecS_F	GGGGACAACTTTGTATACAAAAGTTGCA TAGCACGCTCTTCTTGATGC
	attB4pecS_R	GGGGACAACTTTGTATAGAAAAGTTGGGTG CTAAAGAATCGCCACGTGAGT
	attB4rpecAS_F	GGGGACAACTTTTCTATACAAAGTTGCA CGGCACCATTAATGTATGACTT
	attB3rpecAS_R	GGGGACAACTTTATTATACAAAGTTGT TAGCACGCTCTTCTTGATGC
PiGAPDH (PITG_17130)	gapdh _F	ATATGGCCGGCCCTGCACAACTTTGTCTGCAAG
	gapdh_R	ATATCCTGCAGGAGCACCTCCTTCTCCATCAA
	attB5gapdh S_F	GGGGACAACTTTGTATACAAAAGTTGCA CTGCACAACTTTGTCTGCAAG
	attB4 gapdh S_R	GGGGACAACTTTGTATAGAAAAGTTGGGTG CTAAAGAATCGCCACGTGAGT
	attB4rgapdhAS_F	GGGGACAACTTTTCTATACAAAGTTGCA AGCACCTCCTTCTCCATCAA
	attB3rgapdh AS_R	GGGGACAACTTTATTATACAAAGTTGT CTGCACAACTTTGTCTGCAAG

Supplementary Table S3. Primers for qRT-PCR

Gene	Primer name	Sequence (5´-3´)
ActinA (*P. infestans*) *(PITG_15117.2)*	Actin A-FW<br>Actin A-RV	CATCAAGGAGAAGCTGACGTACA[a]<br>GACGACTCGGCGGCAG[a]
Ubiquitin ligase (P. infestans) (PITG_07230)	Ubil-Fw<br>Ubil-Rev	CTGACAACTTGCCGCTGTATG[b]<br>GCACGAATACCGGAATCGA[b]
PiGPB1 *PITG_06376.1*	Pigpb-Fw<br>Pigpb-Rev	CCGTGAGCATTAACCCACAT<br>AGCTCTCCATACGCTCGAAG
GFP	qGFP- Fw<br>qGFP- R	TATATCATGGCCGACAAGCA<br>CTGGGTGCTCAGGTAGTGGT
Actin (*S. tuberosum*) *embX55749.1*	SActin-Fw<br>SActin-Rev	AAGTACCCGATTGAGCATGG<br>CACTGGCATACAGCGAAAGA
EF-1a (*S. tuberosum*) *(AB061263.1)*	EF-1aFw<br>EF-1aRev	TGAGGCAAACTGTTGCTGTC[c]<br>TGGAAACACCAGCATCACAC[c]
PiO8-3 (*P. infestans*)	PiO8-3-3Fwd<br>PiO8-3-3Rev	CAATTCGCCACCTTCTTCGA[c]<br>GCCTTCCTGCCCTCAAGAAC[c]
PiGAPDH *(PITG_17130)*	gapdhQ_F<br>gapdhQ_R	GCCA TCGTCA TTGACAACAC<br>AGAGTTCGAGA TCGCGTCA T

[a]**Vetukuri RR, Avrova AO, Grenville-Briggs LJ, Van West P, Soderbom F, Savenkov EI, Whisson SC, Dixelius C**. 2011. Evidence for involvement of Dicer-like, Argonaute and histone deacetylase proteins in gene silencing in *Phytophthora infestans*. *Molecular Plant Pathology* **12**, 772-785.

[b]**Judelson HS, Tani S, Narayan RD.** 2009. Metabolic adaptation of *Phytophthora infestans* during growth on leaves, tubers and artificial media. *Molecular Plant Pathology* **10**, 843–855.

[c]**Llorente B, Bravo-Almonacid F, Cvitanich C, Orlowska E, Torres HN, Flawia MM, Alonso GD**. 2010. A quantitative real-time PCR method for in planta monitoring of *Phytophthora infestans* growth. *Letters in Applied Microbiology* **51**, 603-610.

Supplementary Table S4. Primers for hp-RNA expression analysis (Supplementary Figure S3B)

Gene	Primer name	Primer sequence (5´-3´)
PiGPB1	Pig cp_F	TGTGCAGCATTTTCCACCTA
(PITG_06376.1)	Pig cp_R	CTTTCGACGTCCCACAAGAT
PiPEC	Pec cp_F	TGCTTAAGTCGAGCAACGTG
(PITG_02545.1)	Pec cp_R	TTCGCGTACAAAGTGTCCTG
PiCESA2	Ces cp_F	TGGACAACAGCGATGTCATT
(PITG_09964.1)	Ces cp _R	GTTCGGTAGCTCTGCGGTAG
PiGAPDH	Gapdh cp_F	CTGCACAACTTTGTCTGCAAG
(PITG_17130)	Gapdh cp_R	TTCAGCACCTCCTTCTCCAT
GFP	Gfp cp_F	CTGGACGGCGACGTAAAC
	Gfp cp_R	CTAAAGAATCGCCACGTGAGT
EF1a	EF1_F	TGAGGCAAACTGTTGCTGTC
(AB061263.1)	EF1_R	TGGAAACACCAGCATCACAC

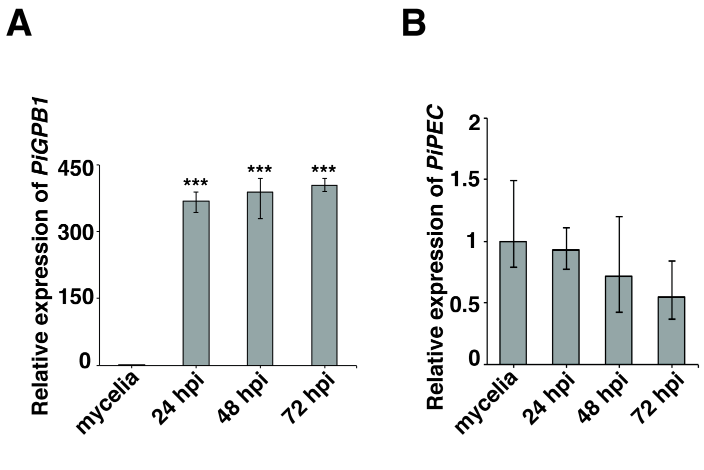

Supplementary Figure S1. Relative *in planta* expression of (A) *PiGPB1* and (B) *PiPEC* gene in wild-type cv. Desiree upon infection with *P. infestans* at 24, 48 and 72 hpi. The expression level of *PiGPB1* and *PiPEC* in non starved mycelia has been assigned the value 1. Data is normalized to *P. infestans actinA* mRNA levels and represent means ± SE (n= 3 pooled leaves of 3 plants). Asterisk indicates significant difference to mycelia (Students' t test; ***p<0.001).

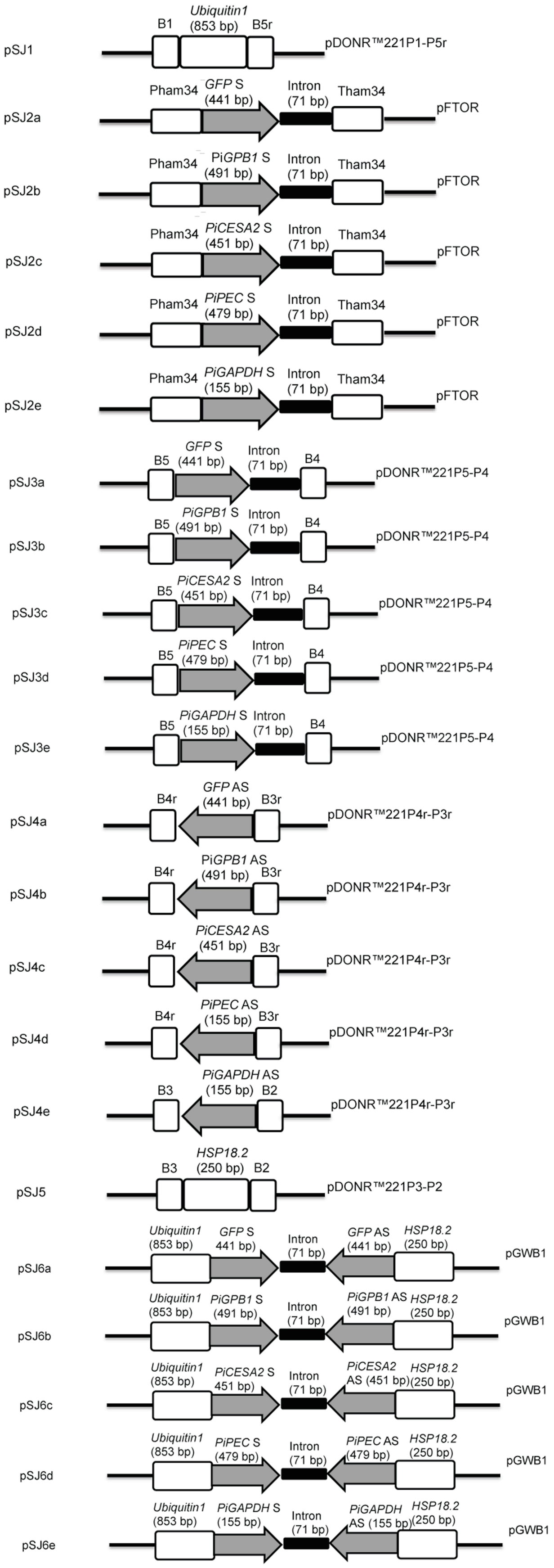

pSJ1
B1
Ubiquitin1
(853 bp)
B5r
pDONR™221P1-P5r
pSJ2a
Pham34
GFP S
(441 bp)
Intron
(71 bp)
Tham34
pFTOR
pSJ2b
Pham34
PiGPB1 S
(491 bp)
Intron
(71 bp)
Tham34
pFTOR
pSJ2c
Pham34
PiCESA2 S
(451 bp)
Intron
(71 bp)
Tham34
pFTOR
pSJ2d
Pham34
PiPEC S
(479 bp)
Intron
(71 bp)
Tham34
pFTOR
pSJ2e
Pham34
PiGAPDH S
(155 bp)
Intron
(71 bp)
Tham34
pFTOR
pSJ3a
B5
GFP S
(441 bp)
Intron
(71 bp)
B4
pDONR™221P5-P4
pSJ3b
B5
PiGPB1 S
(491 bp)
Intron
(71 bp)
B4
pDONR™221P5-P4
pSJ3c
B5
PiCESA2 S
(451 bp)
Intron
(71 bp)
B4
pDONR™221P5-P4
pSJ3d
B5
PiPEC S
(479 bp)
Intron
(71 bp)
B4
pDONR™221P5-P4
pSJ3e
B5
PiGAPDH S
(155 bp)
Intron
(71 bp)
B4
pDONR™221P5-P4
pSJ4a
B4r
GFP AS
(441 bp)
B3r
pDONR™221P4r-P3r
pSJ4b
B4r
PiGPB1 AS
(491 bp)
B3r
pDONR™221P4r-P3r
pSJ4c
B4r
PiCESA2 AS
(451 bp)
B3r
pDONR™221P4r-P3r
pSJ4d
B4r
PiPEC AS
(155 bp)
B3r
pDONR™221P4r-P3r
pSJ4e
B3
PiGAPDH AS
(155 bp)
B2
pDONR™221P4r-P3r
pSJ5
B3
HSP18.2
(250 bp)
B2
pDONR™221P3-P2
pSJ6a
Ubiquitin1
(853 bp)
GFP S
441 bp)
Intron
(71 bp)
GFP AS
(441 bp)
HSP18.2
(250 bp)
pGWB1
pSJ6b
Ubiquitin1
(853 bp)
PiGPB1 S
(491 bp)
Intron
(71 bp)
PiGPB1 AS
(491 bp)
HSP18.2
(250 bp)
pGWB1
pSJ6c
Ubiquitin1
(853 bp)
PiCESA2 S
451 bp)
Intron
(71 bp)
PiCESA2
AS (451 bp)
HSP18.2
(250 bp)
pGWB1
pSJ6d
Ubiquitin1
(853 bp)
PiPEC S
(479 bp)
Intron
(71 bp)
PiPEC AS
(479 bp)
HSP18.2
(250 bp)
pGWB1
pSJ6e
Ubiquitin1
(853 bp)
PiGAPDH S
(155 bp)
Intron
(71 bp)
PiGAPDH
AS (155 bp)
HSP18.2
(250 bp)
pGWB1

Supplementary Figure S2. Schematic representation of plasmid constructs. Constructs (shown in linear form) used for generation of hpRNA in stable transgenic plants. pSJ1 encloses plant promoter *Ubiquitin1* cloned in to pDONR^{TM}221P1-P5r vector. All five constructs pSJ2a, pS2b, pSJ2c, pSJ2d and pSJ2e contain the oomycete ham34 promoter (Pham34) for constitutive expression and terminator (Tham34) and also contain *GFP, PiGPB1, PiCESA2*, *PiPEC*, *PiGAPDH* sequence in sense orientation fused to intron in pFTOR vector. Constructs pSJ3a, pSJ3b, pSJ3c, pSJ3d and pSJ3e contain *GFP, PiGPB1, PiCESA2, PiPEC* and *PiGAPDH* fused with intron in sense orientation and cloned in to pDONR^{TM}221P^{5}-P^{4} vector. Constructs pSJ4a, pSJ4b, pSJ4c, pSJ4d and pSJ4e generated by cloning of *GFP, PiGPB1, PiCESA2, PiPEC* and *PiGAPDH* gene sequence in antisense orientation cloned into pDONR^{TM}221P^{4r}-P^{3r} vector. pSJ5 has plant terminator *HSP18.2*, cloned into pDONR ^{TM}221P^{3}-P^{2} vector. Final constructs pSJ6a, pSJ6b, pSJ6c, pSJ6d and pSJ6e comprise *GFP, PiGPB1, PiCESA2, PiPEC* and *PiGAPDH* gene sequence as inverted repeats with intron I cloned into pGWB1 vector. These five constructs contain *Ubiquitin1* promoter for constitutive expression and *HSP18*.2 terminator. Constructs are not drawn in scale. B1, B5r, B5, B4, B4r, B3r, B3, B2 are B attachment sites of LR clonase. P, promoter; T, terminator; I, 71 bp intron.

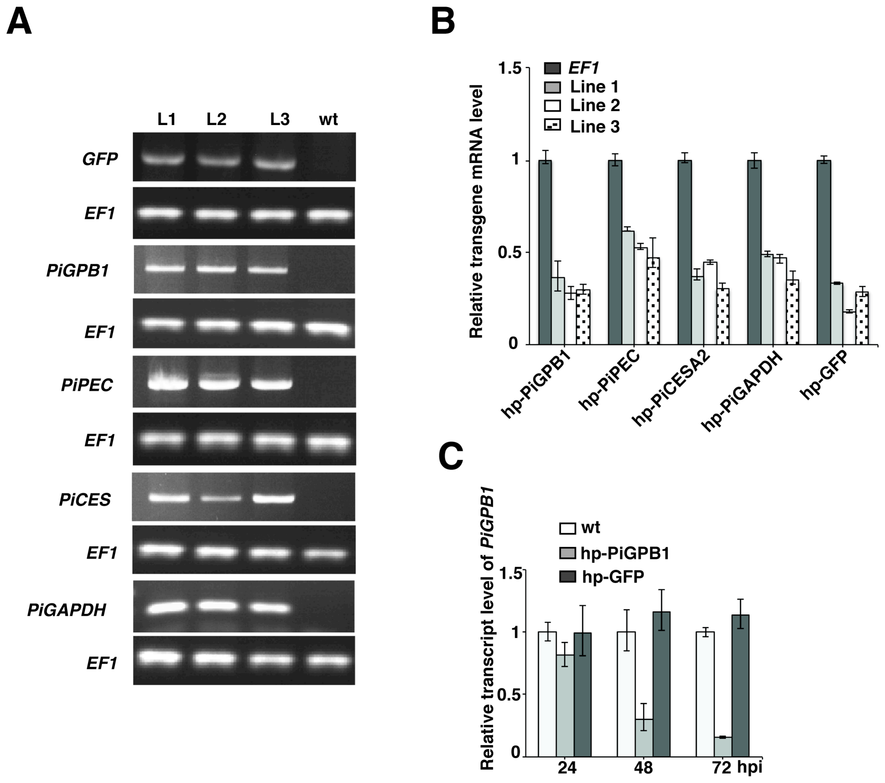

Supplementary Figure S3. Data on hpRNA constructs. (A) PCR amplification of gene of interest from genomic DNA of three individual transgenic lines L1, L2 and L3 of hp-GFP, hp-PiGPB1, hp-PiPEC, hp-PiCESA2 and hp-GAPDH plants. Potato *EF1* is serving as positive control. (B) Expression of hpRNA transgene mRNA level relative to potato *EF1* mRNA level in three individual transgenic lines L1, L2 and L3 of hp-PiGPB1, hp-PiPEC, hp-PiCESA2 hp-PiGAPDH and hp-GFP plants. Data represent means ± SE (n= 3 pooled leaves of 3 plants). (C) Relative transcript level of *PiGPB1* in wild-type (wt), hp-PiGPB1 and hp-GFP plants upon infection with 88069 isolate of *P. infestans* at 24, 48 and 72 hpi. Data is normalized to *P. infestans actinA* mRNA levels and represent means ± SE (n= 3 pooled leaves of 3 plants).

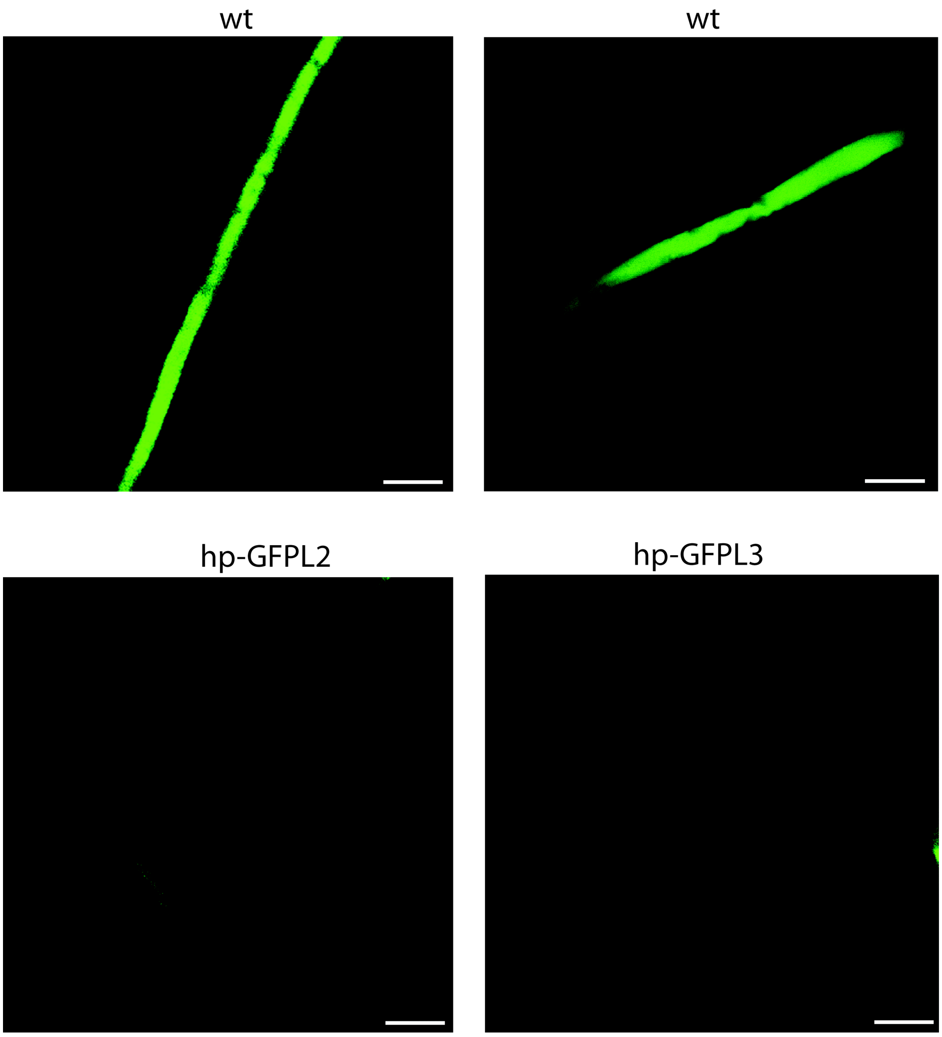

Supplementary Figure S4. Silencing of *GFP* in *P. infestans* by hpRNA. Confocal laser scanning microscopy of *P. infestans* transformants (*Ham34:eGFP*) expressing green fluorescent protein. *GFP* expression in mycelia grown on wild-type (wt) plants (upper panel) and on two individual transgenic lines hp-GFPL2 and hp-GFPL3 (lower panel) at 72 hpi. Bars = 25 μm.

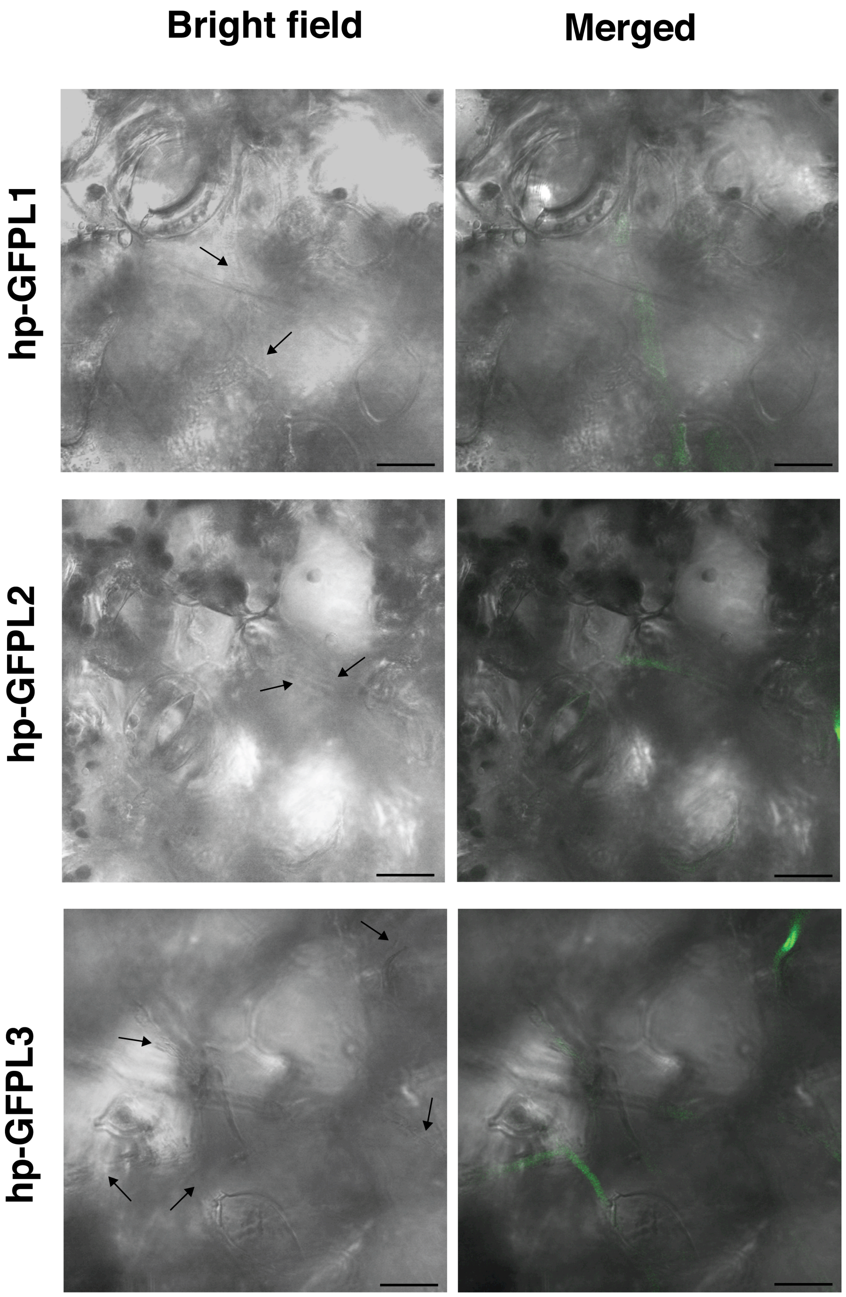

Supplementary Figure S5. Mycelia growth of *GFP*-tagged *P. infestans* on hp-GFP transgenic potato leaves. Bright field microscopy and merged images with fluorescent GFP taken from three individual transgenic lines hp-GFPL1, hp-GFPL2 and hp-GFPL3 at 72 hpi. Bars=25 μm.

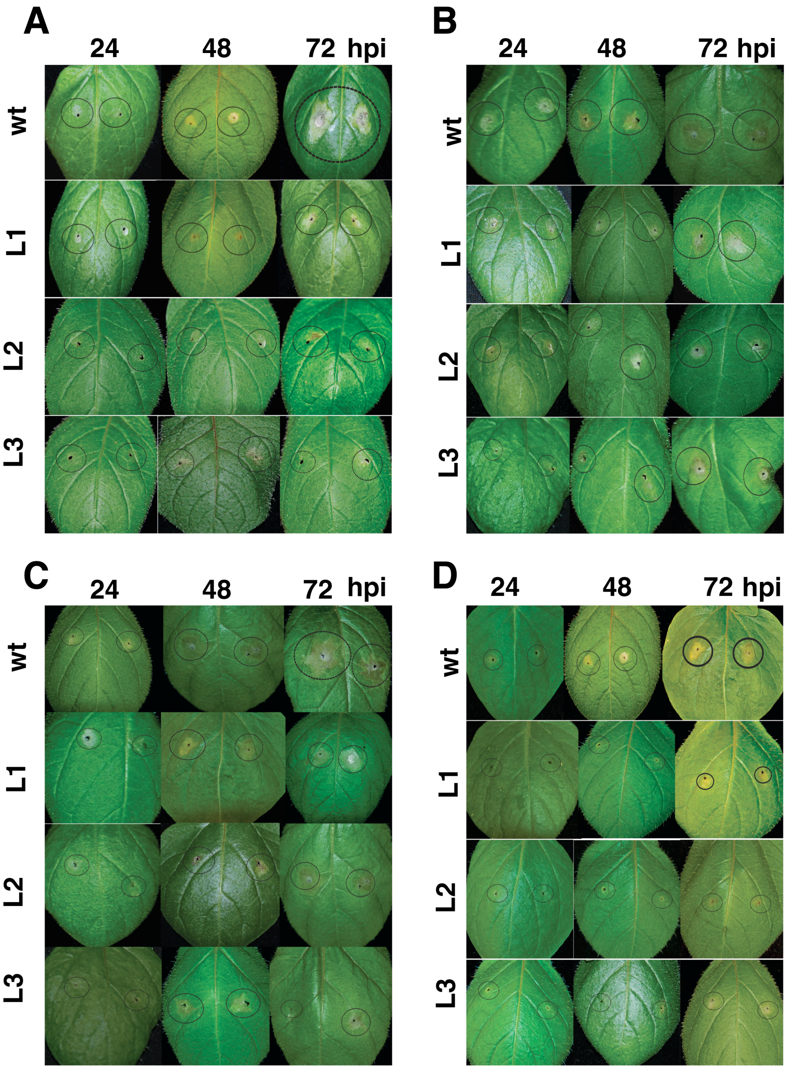

Supplementary Figure S6. Overall picture of necrotic lesions on leaves of wild-type and three individual transgenic lines L1, L2, L3 of (A) hp-PiGPB1 (B) hp-PiPEC (C) hp-PiCESA2 and (D) hp-PiGAPDH plants upon inoculation with *P. infestans* at 24, 48 and 72 hpi.

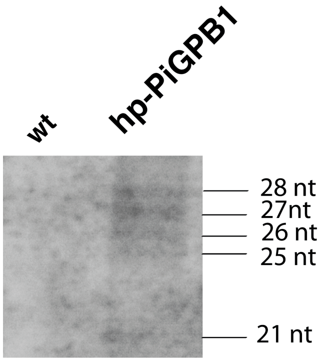

Supplementary Figure S7. Northern blot analysis. Small RNAs complementary to the *PiGPB1* gene was detected in hp-PiGPB1 transgenic plant infected with *P. infestans* at 24 hpi using a *PiGPB1*-specific riboprobe.
